# Identification and validation of prognostic biomarkers related to tumor immune invasion in pancreatic cancer

**DOI:** 10.3389/fgene.2025.1556544

**Published:** 2025-03-10

**Authors:** Minxue Chen, Xinyuan Zhou, Yong Fan, Chen Wang

**Affiliations:** Department of Gastroenterology, Lanzhou University Second Hospital, Lanzhou, China

**Keywords:** CXCL10, CXCL11, pancreatic adenocarcinoma, tumor microenvironment, immunization, biomarker

## Abstract

**Background:**

The diagnosis and treatment of pancreatic adenocarcinoma (PAAD) remain clinically challenging, and new molecular markers for prognostic assessment and targeted therapy are urgently needed. The tumor microenvironment (TME) and immune invasion play an important role in pancreatic cancer development and progression. Therefore, immunotherapeutic strategies based on the TME and immune invasion may have important clinical value.

**Methods:**

In this study, we extracted transcriptome and clinicopathological data for 179 PAAD samples from the TCGA database and evaluated the immune composition, stromal composition, and infiltrating immune cell landscape in the tumor samples. Then, we identified relevant differentially expressed genes (DEGs) and performed functional annotation and prognostic correlation analysis to identify prognostic biomarkers for pancreatic cancer, the correlation between biomarkers and tumor immune invasion was analyzed to reveal the molecular immune mechanism of pancreatic cancer. Finally, GEO databases (GES71729), GEPIA, TISIDB, TIMER databases and RT-PCR were used for further analysis.

**Results:**

CXCL10 and CXCL11 were highly expressed in pancreatic cancer and associated with poor prognosis of patients through cell adhesion molecules chemokine signaling, cytokine-cytokine receptor interaction, natural killer cell-mediated cytotoxicity, and Toll-like receptor signaling pathways. Finally, the correlation between CXCL10 and CXCL11 and tumor immune invasion was analyzed. The results confirmed that the expression levels of CXCL10 and CXCL11 were positively correlated with the contents of CD8^+^ T cells. Activated memory CD4^+^ T cells, M1 macrophages and resting mast cells. The levels of CXCL10 and CXCL11 were related to but negatively correlated with the contents of memory B cells, Tregs and M0 macrophages.

**Conclusion:**

Our study demonstrates that CXCL10 and CXCL11 are novel biomarkers of TME and immune cell infiltration in pancreatic cancer by affecting the distribution of immune cells. CXCL10 and CXCL11 may be new targets for molecular targeted therapy and immunotherapy of pancreatic cancer.

## 1 Introduction

Pancreatic adenocarcinoma (PAAD) is one of the most serious malignant tumors of the digestive system. In the early stage, the only symptoms are upper abdominal or back pain, nausea, abdominal distension and other symptoms, and vascular invasion and distant metastasis occur earlier than in other cancers; thus, most patients lose the optimal opportunity for treatment, and PAAD is thus a serious threat to human health. According to International Agency for Research on Cancer (IARC) statistics, in 2022, 510,992 new cases and 467,409 deaths of PAAD were estimated around the world. PAAD is the 16th most common cancer in estimated new cases and eighth most common cancer in estimated deaths among men and women, with a 5-year survival rate of only 13% (Filho et al., 2025). In addition, the overall morbidity and mortality of PAAD are increasing annually. Estimates indicate that by 2030, pancreatic cancer will have the second highest mortality rate among all malignancies (Park et al., 2021). Although surgical resection alone or combined with radiotherapy and chemotherapy is currently the most effective treatment for localized PAAD, the efficacy is limited (Chiaro et al., 2023). Recently, the new method of immunotherapy has achieved good results in the treatment of malignant tumors, and the search for more accurate tumor immunotherapy targets is the mainly focus of current research (Wang et al., 2021; Fang et al., 2023; Singh and McGuirk, 2020).

Recent years, it has been recognized that the tumor microenvironment (TME) plays an important role in tumor immunity, the TME is a complex internal environmental network composed of various immune cells, extracellular matrix (ECM) components, tumor cells, blood vessels and lymphatic vessels, as well as physical factors. In this network, these complex components are always dynamically changing and are involved in tumor proliferation, angiogenesis, invasion and metastasis as well as in chemotherapeutic resistance and other biological functions (Liu et al., 2023). As the most important components of the TME, immune cells, including activated CD8^+^ T cells, B cells, Macrophages, can cause pathological changes in tissue by inducing tissue fibrosis and stimulating abnormal angiogenesis, eventually leading to the formation of a primary tumor (Rui et al., 2023). Activated CD8^+^ T cells can secrete cytokines and cytotoxic anticancer substances (Durgeau et al., 2018). Macrophages are key mediators of tissue homeostasis, and tumors stimulate macrophage polarization from the M0 phenotype to the proinflammatory M1 phenotype or the tumor-associated M2 phenotype, which promotes tumor proliferation and angiogenesis (DeNardo and Ruffell, 2019). Tumor macrophages have demonstrated the ability to inhibit the recruitment and function of T cells and regulate other aspects of the TME. Furthermore, the TME contains all cytokines, growth factors, chemokines and hormones secreted by stromal cells and tumor cells. These factors can bind to cell surface receptors to activate intracellular downstream signaling pathways in order to regulate various cellular biological behaviors, including tumor immunity. Numerous studies have shown that the TME plays an important role in the response to tumor immunotherapy (Kayla et al., 2021; Du et al., 2020; Lai et al., 2021). However, the mechanism of the TME in PAAD remains unclear.

In this study, the immune/stromal/ESTIMATE (Estimation of Stromal and Immune cells in Malignant Tumors using Expression data) scores for each sample were calculated using the ESTIMATE algorithm [the method provided by Yoshihara et al. (2013)]. We used the CIBERSORT (cell-type identification by estimating relative subsets of RNA transcript) algorithm to analyze the types and correlations of immune cells in the tumor tissue. Then, we identified the relevant differentially expressed genes (DEGs) based on the immune score and stromal score and performed functional annotation and prognostic correlation analyses on the DEGs to identify TME- and prognosis-related biomarkers for pancreatic cancer. Finally, GEO databases (GES71729), GEPIA, TISIDB, TIMER databases and RT-PCR were used to verify the mechanisms of the biomarkers in pancreatic cancer prognosis evaluation and immune infiltration. By analyzing the mechanisms of the biomarkers and immune infiltration, we revealed a potential approach to tumor immunotherapy for PAAD.

## 2 Materials and methods

We used R software (version 3.6.3) (Chen et al., 2021) and Bioconductor (Gentleman et al., 2004) for all statistical analyses throughout our study.

### 2.1 Data collection and processing

Pancreatic cancer-related transcriptome data and clinical data were downloaded from the TCGA database (https://portal.gdc.cancer.gov/). A total of 179 pancreatic cancer samples were included in this study. After collating transcriptome data and extracting clinical data, we used the “limma” (Ritchie et al., 2015) and “estimate” (Yoshihara et al., 2013) packages to calculate the immune score, stromal score, and ESTIMATE score for each sample. The immune and stromal scores were calculated based on the relative proportion of the immune and stromal elements. The ESTIMATE score was calculated as the sum of the immune and stromal scores. For further analysis, we use GEO databases (https://www.ncbi.nlm.nih.gov/geo/, GES71729) and GEPIA (http://gepia.cancer-pku.cn/), TISIDB (http://cis.hku.hk/TISIDB/index.php) and TIMER (https://cistrome.shinyapps.io/timer/) databases to verify the precision of our conclusions.

### 2.2 Correlation analysis of clinical phenotypes and survival

To explore the relationships between target parameters (immune or stromal score, key genes) and clinical characteristics and survival, we analyzed correlations between age, sex (female and male), tumor histological grade (G1, G2, G3, G4), pathological stage (I, II, III, IV), and TNM stage and the three abovementioned scores with the “limma” (Ritchie et al., 2015) and “ggpubr” packages in R. Independent multivariate survival analysis was performed with the “survival” package (Musci et al., 2015) in R, and *P* < 0.05 was considered to indicate a statistically significant difference.

### 2.3 Identification of DEGs

To identify biomarkers associated with the immune and stromal scores, we identified DEGs in this section of the study. We first divided the 179 PAAD samples into high-score and low-score groups based on the median immune and stromal scores. Then, the “limma” (Ritchie et al., 2015) package in R was used for DEG screening, and the “pheatmap” package was used to generate a heatmap of the DEGs. In this study, genes with an adjusted *P* value <0.05 and |log2 fold change (FC) | ≥ 1.0 were considered DEGs.

### 2.4 Gene ontology (GO) and kyoto encyclopedia of genes and genomes (KEGG) enrichment analyses

The “VennDiagram” package was used to obtain the intersections of the upregulated and downregulated genes in the immune score and the stromal score. GO (Ashburner et al., 2000) enrichment analysis and KEGG (Kanehisa and Goto, 2000) pathway analysis were performed with the “Clusterprofiler” package (Yu et al., 2012) in R software to explore the potential functions of the DEGs. GO terms and KEGG pathways for which *P* < 0.05 and *q* < 0.05 were significantly enriched.

### 2.5 Identification of candidate hub genes

Protein-protein interactions (PPIs) were identified using the Search Tool for the Retrieval of Interacting Genes/Proteins (STRING) database (Szklarczyk et al., 2015), and the PPI network was visualized using Cytoscape. The upregulated genes in the high-score group are shown in red, and the upregulated genes in the low-score group are shown in green. The number of nodes in the PPI network was determined. Univariate Cox regression analysis was performed using the R language and “survival” package to identify prognosis-related DEGs with *P* < 0.05 in Kaplan-Meier (KM) analysis and Cox regression analysis.

### 2.6 Gene set enrichment analysis (GSEA)

To identify the functions of hub genes, GSEA (4.0.3) was performed. The 179 PAAD samples were divided into two groups according to the median expression levels of hub genes. We used the gene sets in “c2. cp.kegg.v7.0. symbols.gmt” as the reference gene sets. In this study, KEGG pathways with a nominal *P* < 0.05, false discovery rate (FDR) q-value <0.05, |ES| > 0.5 and FDR <25% were significantly enriched.

### 2.7 Assessment of infiltrating immune cells

The CIBERSORT algorithm was used according to the transcriptome matrix to calculate the contents of immune cells in all PAAD samples, with a *P* value less than 0.05 used as the filtering condition. The “limma” package in R was used to filter the data for further analysis. The data were divided into two groups (high expression group and low expression group) according to the gene expression levels. The “limma” and “vioplot” packages in R were used for differential analysis. In addition, the “ggplot2”, “ggpubr” and “ggExtra” software packages were used for Spearman correlation analysis between gene expression levels and immune cell contents. *P* < 0.05 was considered statistically significant.

### 2.8 Quantitative real-time PCR

The human pancreatic cancer cell lines PANC-1 and AsPC-1 were purchased from CHI Scientific, Inc., MIA PACA-2 and normal pancreatic cancer cell line hTRET-HPNE were purchased from Shanghai Zhongqiaoxinzhou Biotech Company (https://www.zqxzbio.com/). Human pancreatic cancer cell line CFPAC-1 was purchased from the Type Culture Cell Bank of the Chinese Academy of Sciences. All cells were cultured in DMEM (HyClone; Cyvita), supplemented with 10% FBS (Gibco; Thermo Fisher Scientific, Inc.), maintained at 37°C with 5% CO2. Total RNA was extracted from cell lines using Trizol reagent (Invitrogen). Then reverse transcription was performed with a reverse transcription kit (RR037A, Takara Bio, Japan). The expression of CXCL10 and CXCL11 mRNA was detected by LightCycler96 (RR390Q, Takara Bio, Japan) using SYBR Green dye (Takara). The results were analyzed by the method of ΔΔCT. sequences are as follows: CXCL10, Forward: 5′-GTG​GCA​TTC​AAG​GAG​TAC​CTC-3′ and reverse: 5′-TGA​TGG​CCT​TCG​ATT​CTG​GAT​T-3'; CXCL11, Forward: 5′-GAC​GCT​GTC​TTT​GCA​TAG​GC-3′ and reverse: 5′-GGA​TTT​AGG​CAT​CGT​TGT​CCT​TT-3'; GAPDH, forward: 5′-GCA​CCG​TCA​AGG​CTG​AGA​AC-3′ and reverse: 5′-TGG​TGA​AGA​CGC​CAG​TGG​A-3'. All experiments were repeated three times.

### 2.9 Statistical analysis

The Kaplan-Meier method was used to analyze the survival, The chi-squared test was used to compare the categorical variables between the two groups, while continuous variables were compared with Student’s t-test or the Wilcoxon rank sum test for two groups and one-way ANOVA for multiple groups. Univariate and multivariate Cox analyses were used to calculate hazard ratios and 95% confidence intervals. All statistical *P* values are two-sided. *P < 0.05 was considered statistically significant. GraphPad Prism 8.0.1 software were used for statistical analysis.

## 3 Results

### 3.1 The immune score, stromal score and ESTIMATE score were correlated with the clinicopathological characteristics of patients with PAAD

**FIGURE 1:**
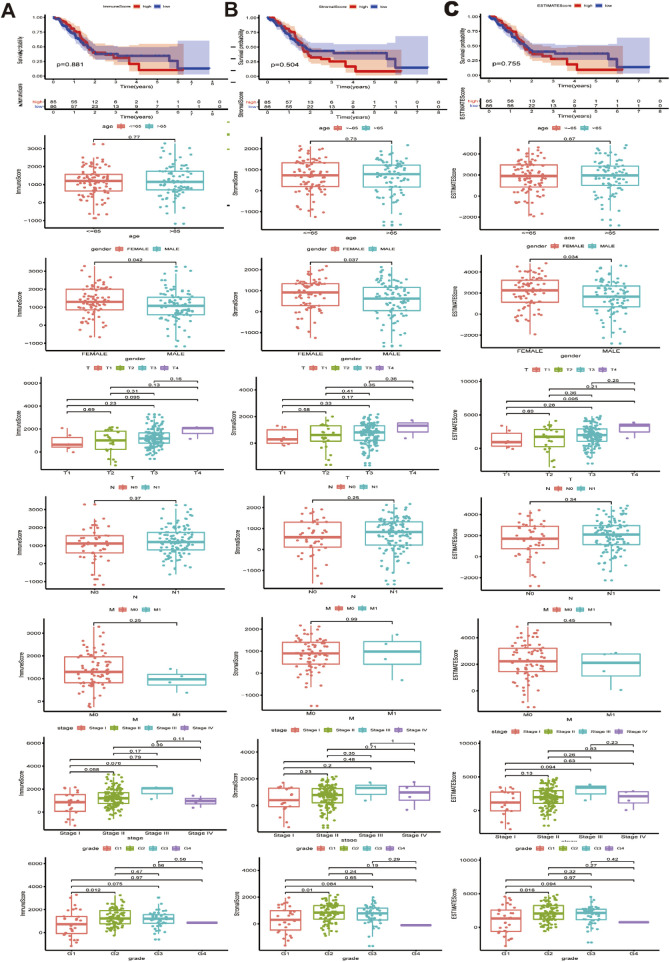
Association between immune/stromal/Estimate score and clinicopathological characteristics. **(A)** Relationship between immune score and survival data, age, gender, TNM stage, stage and pathological stage. **(B)** Relationship between stromal score and survival data, age, gender, TNM stage, stage and pathological stage. **(C)** Relationship between Estimate score and survival data, age, gender, TNM stage, stage and pathological stage. Immune/stromal/Estimate score was significantly associated with gender and G1 and G2 histologically grade (*P* < 0.05).

Transcriptional expression profile and clinical parameter data for 179 PAAD patients in the TCGA cohort were downloaded and integrated. The specific clinicopathological features are shown in Supplementary Table 1. The immune score and stromal score were calculated for each PAAD sample, and the immune score ranged from −1174.89 to 3282.15, the stromal score ranged from −1658.90 to 2170.30, and the comprehensive ESTIMATE score ranged from −2796.19–4831.87. Further analysis showed that the immune score, stromal score and ESTIMATE score were significantly correlated with gender as well as with histological grades G1 and G2 (Figures 1A–C). The *P* values of the associations between the immune, stromal and ESTIMATE scores for sex were 0.042, 0.037 and 0.034, while those for histological grade G1 and G2 tumors were 0.035, 0.02, and 0.038, respectively (Figure 1B). In contrast, no statistical correlations were found between the immune score, stromal score, ESTIMATE score and other clinical and pathological data, such as survival data, age, TNM stage and pathological stage.

### 3.2 CXCL10 and CXCL11 are important prognosis-related DEGs related to the immune score and stromal score

**FIGURE 2:**
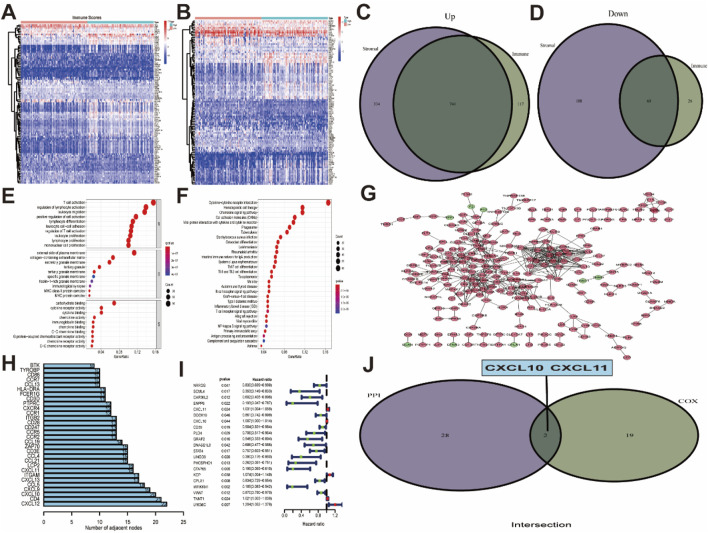
PPI networks combined with univariate Cox regression analysis to identify prognostic genes *relate*d to the immune score and stromal score. **(A, B)** Heat map of differentially expressed genes in the immune score and stromal score. **(C)** Upregulated genes in immune score and stromal score. **(D)** Downregulated genes in immune score and stromal score. **(E, F)** GO functional enrichment analysis and KEGG pathway analysis in 800 commonly DEGs. **(G)** PPI network constructed using all differentially expressed genes, The upregulated genes are shown in red, and the downregulated genes are shown in green. **(H)** Top 30 DEGs sorted by the number of nodes in PPI network. **(I)** The 21 prognostic-related DEGs in Univariate Cox regression analysis. **(J)** CXCL10 and CXCL11were intersectional genes of PPI network construction and univariate Cox regression analysis.

To explore the profiles of DEGs related to the immune and stromal scores, we performed transcriptional microarray analysis of 179 PAAD patients in the TCGA cohort. This analysis was carried out with the “limma” package, and the patients were divided into a high-score group and a low-score group. The 50 genes with the most significant differences were selected for construction of the heat map of DEGs related to the immune and stromal scores (Figures 2A, B). Regarding the immune score, 857 genes were upregulated in the high-score group, and 86 genes were downregulated in the low-score group. Similarly, the group with a high stromal score had 1074 upregulated genes and 248 downregulated genes compared with the group with a low stromal score. The Venn diagram showed that 740 DEGs in the immune score and stromal score were generally upregulated (Figure 2C), while 60 genes were simultaneously downregulated (Figure 2D). In addition, functional enrichment analyses, including GO:BP, GO:CC, GO:MF and KEGG pathway analyses, were performed with these 820 commonly upregulated and downregulated DEGs. After sorting by the log (FDR) values, we listed the top 10 functional ontologies for each section (Figures 2E, F), the DEGs were enriched mainly in immune defense, plasma membrane, cytokine binding, and cytokine-cytokine receptor interactions.

Then, a PPI network was constructed using a filtering condition of 0.95, and the most significant node was identified using Cytoscape software (Figure 2G). The numbers of genes related to the network nodes were determined, and a histogram of the 30 genes related to the most odes was generated (Figure 2H). The results of Cox regression analysis to identify prognosis-related genes among 800 upregulated and downregulated DEGs suggested that 21 genes were closely related to patient prognosis (Figure 2I). Finally, the intersection of the prognosis-related genes and the 30 genes related to the most nodes indicated that the expression of CXCL10 and CXCL11 was significantly related to poor prognosis in PAAD (Figure 2J, *P* < 0.05).

### 3.3 The expression levels of CXCL10 and CXCL11 are correlated with patient prognosis and clinical characteristics and involved in various immune-related signaling pathways

**FIGURE 3:**
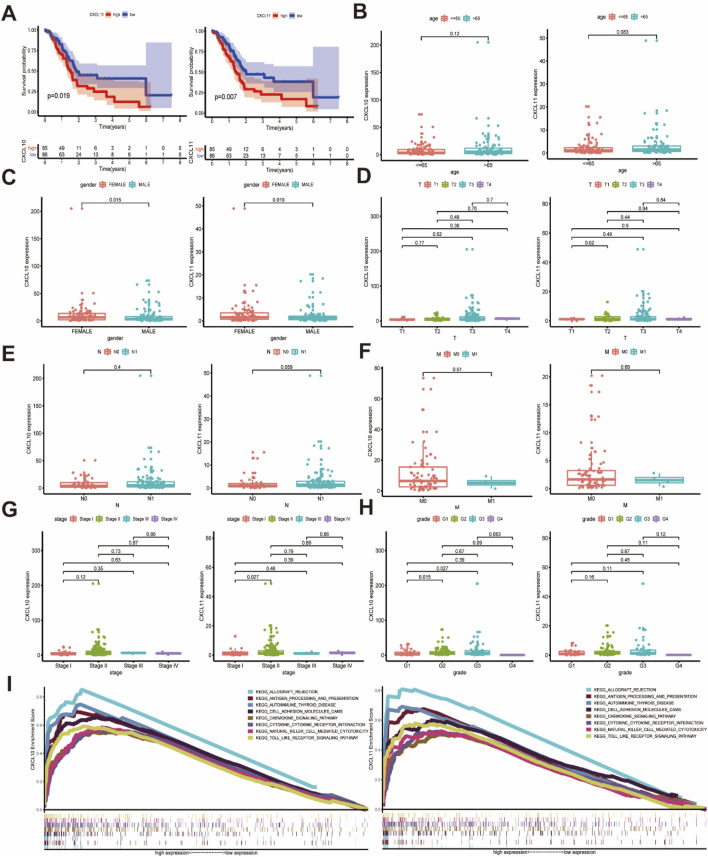
The relationship between the expression of CXCL10 and CXCL11 and the clinicopathological features and the involved mechanism. **(A)** The high expression of CXCL10 and CXCL11 is associated with poor OS in PAAD. **(B)** Relationship between CXCL10 and CXCL11 expression levels and age. **(C)** The expression of CXCL10 and CXCL11 were closely related to gender. **(D–F)** CXCL10 and CXCL11 expression in PAAD was not associated with TNM classification. **(G)** CXCL10 and CXCL11 expression in PAAD with different pathology stage. **(H)** CXCL10 and CXCL11 expression in PAAD with different histologically grade. **(I)** KEGG analysis of multiple GSEAs in CXCL10 and CXCL11.

Based on the mRNA expression profiles and clinical data of CXCL10 and CXCL11 expression in patients, the Kaplan-Meier method was used to analyze the prognosis of patients stratified by CXCL10 and CXCL11 expression levels. Increased mRNA expression levels of CXCL10 and CXCL11 were significantly associated with poor overall survival (OS) (Figure 3A, *P* < 0.05). Correlation analysis of clinical parameters with CXCL10 and CXCL11 expression levels was carried out with the TCGA data. CXCL10 expression was significantly correlated with gender (Figure 3C, *P* = 0.015) and histologically grade (Figure 3H, G1 and G2, *P* = 0.015, G1 and G3, *P* = 0.027) but not with pathology stage (Figure 3G). CXCL11 expression was significantly correlated with gender (Figure 3C, *P* = 0.019) and pathology stage (Figure 3G, stage I and stage II, *P* = 0.027) but not with histologically grade (Figure 3H). The expression levels of CXCL10 and CXCL11 were not statistically significant with patient age and TNM stage, as shown in Figures 3B, D–F.

To further explore the important role of CXCL10 and CXCL11 in pancreatic cancer, we analyzed the related signaling pathways involving CXCL10 and CXCL11 through GSEA. The results suggested that the signaling pathways involving CXCL10 and CXCL11 were consistent and related. The KEGG pathway diagram from the results of multiple GSEAs for the pathway with the strongest correlation was drawn (Figure 3I, FDR q-val <0.05), and the specific parameters of each signaling pathway are listed in Supplementary Table 2. CXCL10 and CXCL11 were involved in multiple signaling pathways, such as allograft rejection, antigen processing and presentation, autoimmune thyroid disease, cell adhesion molecules (CAMs), chemokine signaling, cytokine-cytokine receptor interaction, natural killer (NK) cell-mediated cytotoxicity, and Toll-like receptor signaling, suggesting that CXCL10 and CXCL11 are involved in the pancreatic cancer TME and play an important role in immune infiltration.

### 3.4 Association of CXCL10 and CXCL11 with immune cell infiltration

**FIGURE 4:**
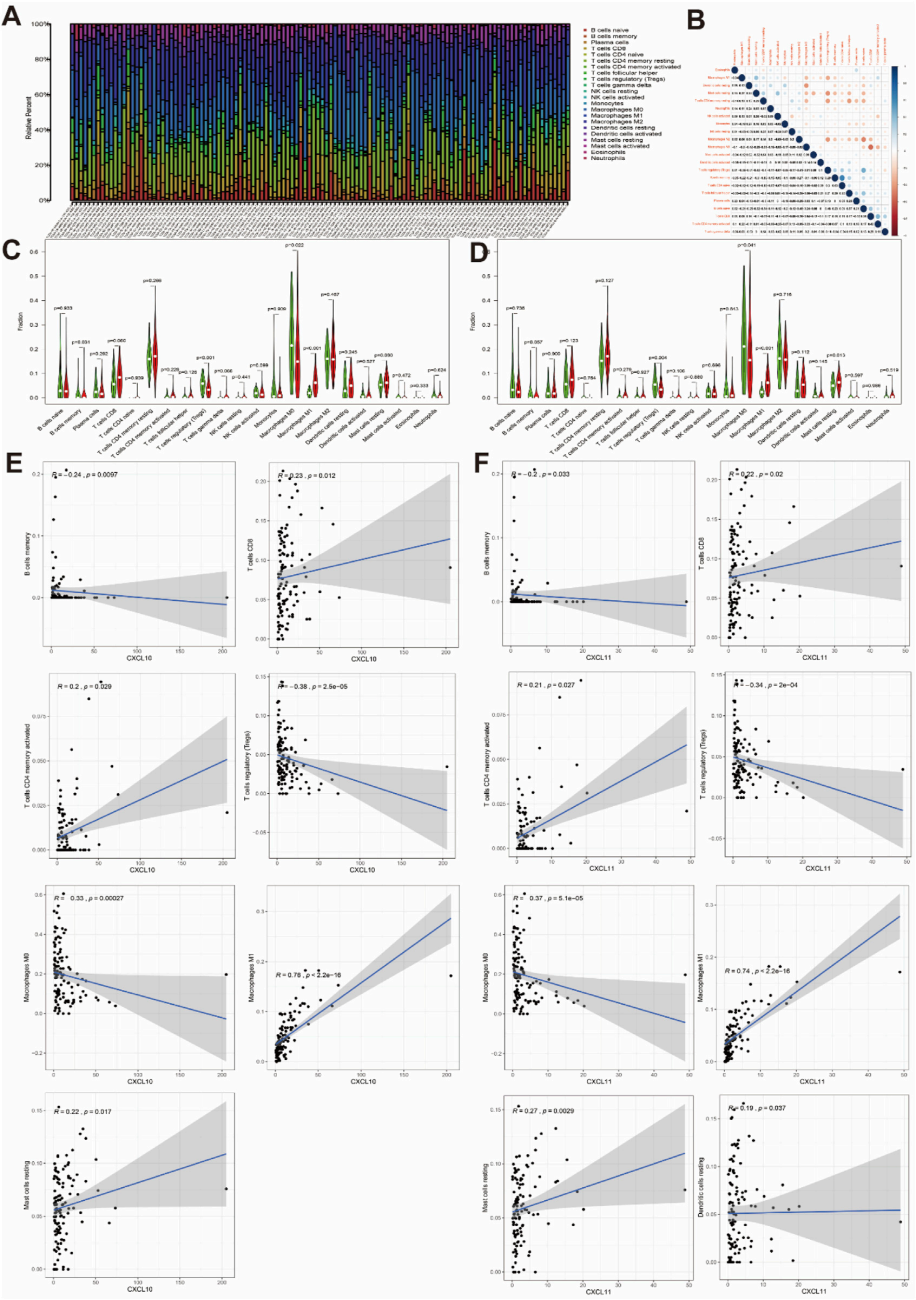
Correlation of CXCL10 and CXCL11 expression with immune infiltration in PAAD. **(A)** Distribution of 22 immune cells in 116 PAAD samples. **(B)** Correlation between 22 kinds of immune cells. The number and size of the circle on behalf of the correlation value between the corresponding 2 cells. **(C)** Correlation of 22 kinds of immune infiltrating cells in high and low CXCL10 expression groups. **(D)** Correlation of 22 kinds of immune infiltrating cells in high and low CXCL11 expression groups. The red violin diagram represents the high expression group and the green violin diagram represents the low expression group. **(E)** Correlation between infiltrating immune cells and the expression of CXCL10. **(F)** Correlation between infiltrating immune cells and the expression of CXCL11.

In addition to analyzing the relationship between immune score and interstitial score and clinicopathological features and DEGs in pancreatic cancer patients, we also analyzed the immune cell content in each tumor sample by excluding genes with expression levels below zero in the normal and tumor samples. The cells in the 116 samples were closely related to tumor immunity (*P* < 0.05), and a total of 22 types of infiltrating immune cells were identified, including T cells, B cells, NK cells, and lymphocytes (Figure 4A). We then analyzed the correlations among these 22 immune cell types (Figure 4B). After confirming the clinical value of CXCL10 and CXCL11, we analyzed the differences in expression and correlations between the expression levels of CXCL10 and CXCL11 and the contents of immune cells. Analysis of the contents of the two groups of immune cells with high and low gene expression suggested that expression of CXCL10 was significantly related to the contents of memory B cells, regulatory T cells (Tregs), M0 macrophages, and M1 macrophages, while the expression of CXCL11 was significantly related to the contents of Tregs, M0 macrophages, M1 macrophages and resting mast cells (Figures 4C, D, *P* < 0.05). Scatter plots were then generated to show partial Spearman correlation data and statistical significance. The results suggested that the expression levels of CXCL10 and CXCL11 were positively correlated with the contents of CD8^+^ T cells, activated memory CD4^+^ T cells, M1 macrophages and resting mast cells. The levels of CXCL10 and CXCL11 were related to but negatively correlated with the contents of memory B cells, Tregs and M0 macrophages. In addition, the expression level of CXCL11 was positively correlated with the content of resting dendritic cells (Figures 4E, F). Combining the conclusions of the differential and correlation analyses, we concluded that memory B cells, T-cells regulatory (Tregs), M0 macrophages, and M1 macrophages are CXCL10-related immune cells, while Tregs, M0 macrophages, M1 macrophages, and resting mast cells are CXCL11-related immune cells (Table 1). T-cells regulatory (Tregs), M0 macrophages, and M1 macrophages are co-owned by CXCL10 and CXCL11, suggesting that they can provide help in tumor immunotherapy of PAAD.

**TABLE 1:** The intersection of difference analysis and correlation analysis.

Tumor infiltrated immune cells	CXCL10		CXCL11	
	*P*_CT_	*P*_DT_	*P*_CT_	*P*_DT_
B-cells memory	0.01	0.03	-	-
T-cells regulatory	0.00	0.00	0.00	0.00
macrophages M0	0.00	0.02	0.00	0.04
macrophages M1	0.00	0.00	0.00	0.00
Mast cells resting	-	-		0.01

*P*_CT_: Correlation test (*p*-value). *P*_DT_: Difference test (*p*-value).

### 3.5 Validation of CXCL10 and CXCL11 related to pancreatic cancer tumor prognosis and immune invasion

**FIGURE 5:**
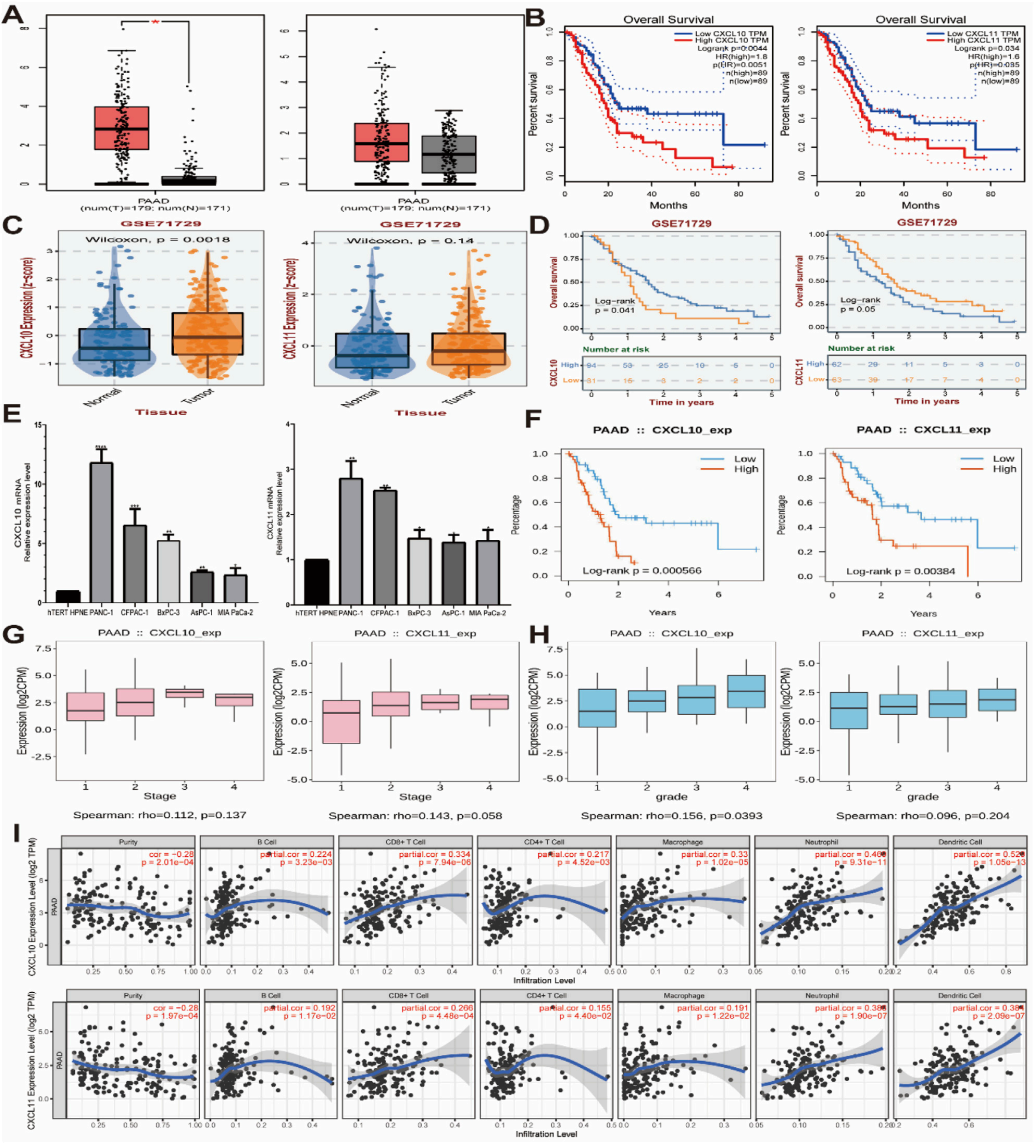
mRNA expression levels of CXCL10 and CXCL11 in pancreatic cancer cell lines. **(A, C)** CXCL10 and CXCL11 mRNA relative expression level in GEPIA and GEO databases (GES71729). **(B, D)** The survival analysis of CXCL10 and CXCL11 in GEPIA and GEO databases (GES71729). **(E)** CXCL10 and CXCL11 mRNA relative expression level in pancreatic cancer cell lines. **(F)** The survival analysis of CXCL10 and CXCL11 in TISIDB databases. **(G, H)** The stage and grede analysis of CXCL10 and CXCL11 in TISIDB databases. **(I)** The immune infiltrating cells analysis of CXCL10 and CXCL11 in TIMER databases.

In summary, we have identified CXCL10 and CXCL11 as key genes affecting the prognosis and immune infiltration of pancreatic cancer. For further validation, we used GEPIA and GEO databases (GES71729) to verify the expression of CXCL10 and CXCL11 in pancreatic cancer and their relationship with the prognosis of patients. The results showed that the expression of CXCL10 and CXCL11 was significantly increased in pancreatic cancer, and CXCL10 was statistically significant (Figures 5A, C). The higher the expression of CXCL10 and CXCL11, the worse the prognosis (Figures 5B, D). Subsequently, we conducted RT-PCR detection in multiple pancreatic cell lines, and the results indicated that the expression levels of CXC10 and CXCL11 in pancreatic cancer cell lines were significantly increased, the differences were statistically significant (Figure 5E), and the expression trends of PANC-1, CFPAC-1 and BxPC-3 were identical. However, the expression of CXCL10 in AsPC-1 was higher than that of MIA PaCa-2, while CXCL11 was the opposite. The prognostic data in TISIDB database further validated our results (Figure 5F), and CXCL10 expression level was correlated with grade (Figures 5G, H). Finally, we used TIMER database to analyze the relationship between CXCL10 and CXCL11 and immune cells, suggested that the expression of CXCL10 and CXCL11 is correlated with CD8^+^ T cells, CD4^+^ T cells, macrophages and B-cells (Figure 5I), Cox Proportional Hazard Model showed that age, CD4^+^ T cells, Neutrophil, Dendritic cells, CXCL10 and CXCL11 were risk factors affecting the prognosis of patients (Table 2), which was consistent with our previous results. To sum up, CXCL10 and CXCL11 as Prognostic Biomarkers and Mediators of Tumor Immune Infiltration in Pancreatic Adenocarcinoma.

**TABLE 2:** Cox proportional hazard model.

Type	CXCL10				CXCL11			
	HR	95%CI _l	95%CI _u	*P*	HR	95%CI _l	95%CI _u	*P*
Age	1.02	1.00	1.04e+00	0.049*	1.03	1.00	1.05e+00	0.023*
Gender	0.91	0.59	1.40e+00	0.65	0.89	0.58	1.38e+00	0.611
stage2	1.23	0.54	2.79e+00	0.62	1.15	0.51	2.61e+00	0.735
stage3	0.39	0.05	3.25e+00	0.39	0.41	0.05	3.41e+00	0.410
Stage4	0.68	0.14	3.35e+00	0.632	0.68	0.14	0.137	0.634
Purity	0.40	0.16	1.01e+00	0.052	0.42	0.17	1.08e+00	0.071
B_cell	309.75	0.83	1.16e+05	0.058	103.70	0.34	3.14e+04	0.111
CD8_Tcell	13.81	0.03	6.28e+03	0.400	19.81	0.05	7.61e+03	0.32
CD4_Tcell	0.00	0.00	1.06e-01	0.011*	0.00	0.00	3.29e-01	0.024*
Macrophage	0.02	0.00	1.60e+01	0.261	0.02	0.00	1.43e+01	0.249
Neutrophil	2022418.92	1.49	2.75e+12	0.044*	1394385.51	1.20	1.63e+12	0.047*
Dendritic	0.00	0.00	1.93e-01	0.006**	0.01	0.00	4.63e-01	0.018*
CXCL10	1.32	1.10	1.59e+00	0.003**				
CXCL11					1.28	1.06	1.54e+00	0.009**

HR: hazard ratio. 95%CI_l: lower 95% confidential interval. 95%CI_u: upper 95% confidential interval.

The meaning of the bold values indicates that the P values are statistically significant.

## 4 Discussion

Pancreatic cancer is a disease that seriously endangers human health, and methods for its early diagnosis and treatment are limited, resulting in poor prognosis (Mizrahi et al., 2020). Recent research on pancreatic tumor biology has focused on the TME (Hessmann et al., 2020; Karamitopoulou, 2019; Meng et al., 2020), and an increasing number of studies have shown that the TME plays an important role in the occurrence and development of pancreatic cancer and tumor immunotherapy. Immunotherapy has achieved great results, but its efficacy is limited. Moreover, the molecular pathways and potential mechanisms of the TME in pancreatic cancer have not been fully elucidated (Ho et al., 2020), and new comprehensive treatment strategies are urgently needed.

In our study, transcriptome expression data (ESTIMATE algorithm) of PAAD patients were used to evaluate the immune score, stromal score, and ESTIMATE score for each tumor sample. The immune score, stromal score and comprehensive score were closely related to the clinicopathological characteristics of patients with pancreatic cancer. Subsequently, we evaluated the DEGs related to the immune score and the stromal score and determined the prognosis-related genes from the clinicopathological data, and we identified CXCL10 and CXCL11 as prognostic factors in the pancreatic cancer TME. Finally, the molecular mechanism by which CXCL10 and CXCL11 are involved in immune cell infiltration in pancreatic cancer was analyzed, and these results are anticipated to facilitate pancreatic cancer immunotherapy.

CXCL10 and CXCL11 are ELR-negative CXC chemokines that participate in the chemotaxis, differentiation and activation of peripheral immune cells by binding to the CXCR3 receptor (Tokunaga et al., 2018; Karin, 2020). Some studies have shown that CXCL10 and CXCL11 can inhibit angiogenesis and exert antitumor effects (Keeley et al., 2010). However, CXCL10 and CXCL11 can also increase tumor proliferation and metastasis (Boye et al., 2017). Recent studies have explored the role of CXCL10 and CXCL11 in pancreatic cancer. CXCL10 is overexpressed in human pancreatic cancer and is related to the poor survival of patients with PAAD (Delitto et al., 2015). The serum CXCL10 level in pancreatic cancer patients with lymph node metastasis is significantly higher than that in pancreatic cancer patients without lymph node metastasis (Jiang et al., 2013). CXCL10 can promote the migration of pancreatic cancer cells to sensory neurons and mediate the pain response in patients (Hirth et al., 2020). Interferon-γ (IFNγ) is the key cytokine in antitumor immunity and can affect a variety of cells in the TME of pancreatic cancer (Delitto et al., 2015), Although the study of Huimin Huang demonstrated the role of CXCL10 in the immune infiltration of PAAD (Huang et al., 2021), it was not comprehensive enough in the prognostic biomarkers and immune infiltration mechanism, and the research content was relatively limited. Our study has detailly demonstrated the synergistic mechanism of CXCL10 and CXCL11 in the ELR-negative CXC chemokines gene family in the tumor microenvironment and immune infiltration of PAAD. Most importantly, the GEO databases (GES71729), GEPIA, TISIDB, TIMER databases PAAD cohort were used to verify our results, the expression levels of CXCL10 and CXCL11 in pancreatic cancer and normal pancreatic epithelial cells were verified by RT-PCR. which is more convincing and has potential research value, and further studies are needed. Furthermore, the research on CXCL11 in pancreatic cancer is still in the initial stage, related studies have only shown that CXCL11 is highly expressed in the serum of patients with pancreatic cancer and has a protumor function (Torres et al., 2014; Ge et al., 2020). Nevertheless, our research confirmed that CXCL10 and CXCL11 are important genes for the prognostic evaluation of pancreatic cancer. Most importantly, our study explored the mechanism of action of CXCL10 and CXCL11 in the TME and immune cell infiltration of PAAD and provided information anticipated to facilitate pancreatic cancer immunotherapy.

The composition and function of tumor-infiltrating immune cells are changed according to the immune status of the host (Shibutani et al., 2018; Udall et al., 2018). Tumor-associated macrophages (TAMs) are the most abundant immune cells infiltrating the TME. In the early stage of tumor development, tumor-infiltrating M1-polarized macrophages usually show a phenotype of high IL-12 and low IL-10 expression, promoting the immune response and causing lysis of tumor cells. During the development of advanced tumors, TAMs are usually polarized toward the M2 phenotype, promote tumor invasion and metastasis, and create a favorable microenvironment promoting tumor survival, growth and angiogenesis (Pathria et al., 2019; Zhu et al., 2021). The results of this study showed that the expression levels of CXCL10 and CXCL11 were negatively correlated with the level of M0 macrophages, positively correlated with the level of M1 macrophages and not correlated with the level of M2 macrophages, possibly because most of the PAAD samples included in this study were from tumors in the early stage of development, and M0 macrophages inhibit tumor-specific T cell immunity and enhance tumor growth. Or there is immune escape of M1 macrophages, which leads to the failure of M1 macrophages to exert their inhibition of tumor growth and tumor-induced angiogenesis. Therefore, further study of M1 macrophage immune escape mechanism is helpful for the treatment of PAAD.

Some studies have shown that B cells can induce and maintain beneficial antitumor activity, while others have found that B cells may play a protumor role due to their different immunosuppressive subtypes (Wang et al., 2019). The results of this study showed that the content of memory B cells was inversely proportional to the expression levels of CXCL10 and CXCL11, indicating that the antitumor activity of B cells was inhibited in PAAD. CD8^+^ T cells, as one of the main antitumor effector immune cells, are activated by signaling from specific dendritic cells with the help of CD4^+^ T cells to CD8^+^ T cells in order to optimize the scale and quality of the cytotoxic T lymphocyte (CTL) response (Borst et al., 2018), but this study showed that the contents of these cells are proportional to the CXCL10 and CXCL11 expression levels. This relationship might be explained by the observation that when CD8^+^ T cells infiltrate into tumor tissue, they are usually in a dysfunctional state characterized by impaired activation and proliferation abilities; thus, the apoptosis rate is increased and the effect of cytokine production is reduced (He et al., 2019). These dysfunctional CD8^+^ T cells are a barrier to successful cancer elimination. As immune suppressor cells, Tregs play a negative immunoregulatory role in the immune response and can inhibit the proliferation and activation of T cells (Yan et al., 2019). However, in the current study, we found that the CXCL10 and CXCL11 expression level was negatively correlated with the Treg content, Lunardi S et al. 's study show CXCL10 could recruit CD4+/CD8+ effector T cells and FoxP3C- Tregs, however, due to the large increase in circulating Tregs compared with effector T cells, CXCR3C Tregs may be preferentially recruited to inhibit the adaptive immune response (via effector T cells and NK cells) (Lunardi et al., 2015), thus establishing an immunosuppressive and protumor microenvironment that promotes poor prognosis in cancerous person (Tanaka and Sakaguchi, 2017), which contradicted with our results, suggesting that Tregs may have more complex immunomodulatory mechanisms that may be explained by the combination of specific immune-related signaling pathways of CXCL10 and CXCL11. At the present, all the studies on CXCL10 and CXCL11 are limited to the study of cells, CDX and PDX model and other basic models. However, tumor immunity is an anti-tumor process involving multiple Spaces and dimensions. In the future, CXCL10 and CXCL11 knockout mice may be used to further verify the mechanism of CXCL10 and CXCL11 in tumor immune cell infiltration of pancreatic cancer, so as to further study the maintenance or restoration of tumor infiltration efficiency of CD8^+^ T cells and assist the development of tumor-specific CTLs in lymphoid organs. Establishing effective and durable anti-tumor immunity or combining targeted Treg cells with the activation of tumor-specific effector T cells can help in the development of new therapeutic strategies to improve the immunotherapy effect of PAAD.

## 5 Conclusion

Our date showed that the immune score, stromal score and ESTIMATE score were significantly correlated with the sex and grade of pancreatic cancer patients. The results of the PPI network and Cox regression analyses showed that CXCL10 and CXCL11 are valuable factors involved in the TME of pancreatic cancer and are correlated with the prognosis and clinicopathological characteristics of pancreatic cancer patients. In addition, we revealed the relationship of CXCL10 and CXCL11 in immune cell infiltration landscape of pancreatic cancer. Our findings indicate that CXCL10 and CXCL11 may be new targets for pancreatic cancer immunotherapy. However, our study has some limitations; it was limited to bioinformatic predictions and the mechanism of immune infiltration is not well defined, further studies are needed.

## Data Availability

The data presented in the study are deposited in the GitHub (https://github.com/MinxueChen/CXCL10-and-CXCL11/tree/master) repository, accession number MinxueChen, password Cmx199409062339.
