# Supplementary material for: Identification and validation of prognostic biomarkers related to tumor immune invasion in pancreatic cancer
Source: Front Genet. 2025 Mar 10;16:1556544. doi: 10.3389/fgene.2025.1556544 (PMC11931078; doi:10.3389/fgene.2025.1556544)
Supplement: Supplementary file 1 [file Table1.docx]

**SUPPLEMENTARY MATERIAL**

**Identification and validation of prognostic biomarkers related to tumor immune invasion in pancreatic cancer**

Minxue Chen1, Xinyuan Zhou1, Yong Fan1, Chen Wang1*

1 Department of Minimally Invasive Surgery, The Second Hospital of Lanzhou University, Lanzhou 730000, China.

***Correspondence** **author:** Chen Wang

Email addresses: [chenwang@lzu.edu.cn](mailto:chenwang@lzu.edu.cn)

Supplementary Table 1. Clinico-pathological characteristics statistics of PAAD patients from TCGA database

| Clinical characteristics |  | | Total | | % |
| --- | --- | --- | --- | --- | --- |
| Age at diagnosis (year)  Gender  Grade  Stage  T classification  M classification  N classification | <=65  >65  Female  Male  G1  G2  G3  G4  Ⅰ  Ⅱ  Ⅲ  Ⅳ  T1  T2  T3  T4  M0  M1  N0  N1 | 91  88  81  98  31  95  50  1  20  147  4  5  6  23  142  4  81  5  48  127 | | 50.84  49.16  45.25  54.75  17.51  53.67  28.25  0.57  11.36  83.52  2.27  28.51  3.43  13.14  81.14  2.29  94.19  5.81  27.43  72.57 | |

Supplementary Table 1. Enriched signaling pathway sets

| Kyoto Encyclopedia of Genes and Genomes name | CXCL10 | | | CXCL11 | | |
| --- | --- | --- | --- | --- | --- | --- |
|  | NES | NOM p-val | FDR q-val | NES | NOMp-val | FDR q-val |
| KEGG_ALLOGRAFT_REJECTION | 2.00 | 0.00 | O.00 | 1.91 | 0.00 | 0.01 |
| KEGG_ANTIGEN_PROCESSING_AND_PRESENTATION | 2.21 | 0.00 | 0.OO | 2.12 | 0.00 | 0.01 |
| KEGG_AUTOIMMUNE_THYROID_DISEASE | 2.11 | 0.00 | 0.00 | 1.97 | 0.01 | 0.01 |
| KEGG_CHEMOKINE_SIGNALING_PATHWAY | 1.98 | 0.00 | 0.01 | 1.82 | 0.01 | 0.03 |
| KEGG_CYTOKINE_CYTOKINE_RECEPTOR_INTERACTION | 2.02 | 0.00 | 0.01 | 1.91 | 0.00 | 0.01 |
| KEGG_CELL_ADHESION_MOLECULES_CAMS | 2.22 | 0.00 | 0.00 | 2.02 | 0.00 | 0.01 |
| KEGG_NATURAL_KILLER_CELL_MEDIATED_CYTOTOXICITY | 2.04 | 0.00 | 0.00 | 1.85 | 0.01 | 0.03 |
| KEGG_TOLL_LIKE_RECEPTOR_SIGNALING_PATHWAY | 2.11 | 0.00 | 0.00 | 2.06 | 0.00 | 0.01 |

NES: normalized enrichment score. NOM: nominal p-value. FDR: False Discovery Rate. Gene sets with NOM p-value and FDR q-val less than 0.05 was considered as statistical significance.
